# VEGF and EGFR signaling pathways are involved in the baicalein attenuation of OVA-induced airway inflammation and airway remodeling in mice

**DOI:** 10.1186/s12931-023-02637-6

**Published:** 2024-01-04

**Authors:** Wang Peng, Qinxuan Xia, Yue Zhang, Danfeng Cao, Xiangrong Zheng

**Affiliations:** 1 Department of Pediatrics, Xiangya Hospital, Central South University, 87 Xiangya Road, Changsha, Hunan 410008 China; 2 Department of Clinical Pharmacology, Xiangya Hospital, Central South University, 87 Xiangya Road, Changsha, Hunan 410008 China; 3 Respiratory and critical care medicine, Xiangya Hospital, 87 Xiangya Road, Changsha, Hunan 410008 China; 4 Academician Workstation and The Hunan Provincial University Key Laboratory of the Fundamental and Clinical Research on Functional Nucleic Acid, Changsha Medical University, Changsha, Hunan 410219 China; 5 Department of Pharmacy, Union Hospital, Tongji Medical College, Huazhong University of Science and Technology, No. 1227, Jiefang Road, Wuhan, Hubei 430022 China

**Keywords:** Asthma, Baicalein, Traditional Chinese Medicine, Network pharmacology

## Abstract

**Background:**

Although Traditional Chinese Medicine (TCM) has been used for treating asthma for centuries, the understanding of its mechanism of action is still limited. Thus, the purpose of this study was to explore the possible therapeutic effects, and underlying mechanism of baicalein in the treatment of asthma.

**Methods:**

Freely availabled atabases (e.g. OMIM, TTD, Genecards, BATMAN-TCM, STITCH 5.0, SEA, SwissTargetPrediction) and software (e.g. Ligplot 2.2.5 and PyMoL) were used for disease drug target prediction and molecular docking by network pharmacology. The efficacy and mechanism of action of baicalein in the treatment of asthma were validated using an ovalbumin (OVA)-induced asthma mouse model and molecular biology techniques.

**Results:**

A total of 1655 asthma-related genes and 161 baicalein-related targets were identified from public databases. Utilizing common databases and software for network pharmacology and molecular docking analysis, seven potential target proteins for the therapeutic effects of baicalein on asthma were selected, including v-akt murine thymoma viral oncogene homolog 1 (AKT1), vascular endothelial growth factor A (VEGFA), epidermal growth factor receptor (EGFR), proto-oncogene tyrosine-protein kinase Src (SRC), mitogen-activated protein kinase 3 (MAPK3), matrix metallopeptidase 9 (MMP9), and MAPK1. In vivo, baicalein treatment via intraperitoneal injection at a dose of 50 mg/kg significantly reduced airway inflammation, collagen deposition, smooth muscle thickness, lung interleukin (IL)-4 and IL-13 levels, peripheral blood immunoglobulin (Ig)E levels, as well as the count and ratio of eosinophils in bronchoalveolar lavage fluid (BALF) in an OVA-induced asthma mouse model. Further validation by reverse transcription quantitative polymerase chain reaction (RT-qPCR) and western blotting analysis revealed that the VEGF and EGFR signaling pathways involving VEGFA, MAPK1, MAPK3, and EGFR were inhibited by baicalein in the asthma mouse model.

**Conclusion:**

Baicalein attenuates airway inflammation and airway remodeling through inhibition of VEGF and EGFR signaling pathways in an OVA-induced asthma mouse model. This will provide a new basis for the development of baicalein as a treatment for asthma and highlights the potential of network pharmacology and molecular docking in drug discovery and development.

**Supplementary Information:**

The online version contains supplementary material available at 10.1186/s12931-023-02637-6.

## Introduction

Asthma is a complex respiratory disorder that is characterized by persistent airway inflammation and remodeling, which often causes coughing, wheezing, shortness of breath and chest tightness. Recent data show that nearly 262 million people suffered from asthma, and that 461,000 of these died in 2019 [1].

To date, asthma remains an incurable disease, with current treatment goals aiming to achieve good symptom control, including minimizing the risk of asthma-related mortality, exacerbations, persistent airflow limitation and medication-related side effects [2]. Corticosteroids are known to effectively control asthma, as they suppress inflammation, edema, and mucus hypersecretion [3]. However, long-term or high-dose use of glucocorticoids may lead to serious complications, such as osteoporosis [4], hyperglycemia [5], and infections caused by immune system suppression [6]. Hekking et al. showed that approximately 24% of asthmatics requiring moderate to high doses of inhaled corticosteroids [7]. One of the possible reasons is the inadequate expression of glucocorticoid receptors in patients with severe asthma [8]. Therefore, there is an unmet need for alternative therapies to reduce the collateral systemic effects of steroid use, or to treat severe corticosteroid-insensitive asthma. Possibilities include the development and use of traditional Chinese medicine (TCM), acupuncture and breathing function exercises [9].

*Scutellaria baicalensis Georgi* is a plant whose roots are commonly used in TCM, containing over 30 kinds of flavonoids, including baicalin, baicalein, and wogonin, which have very broad spectra of biological applications, including use as anti-inflammatory [10], anti-oxidant [10], anti-bacterial [11] and antiallergic [12] agents. Notwithstanding their wide use, very little is understood about how these flavonoids function so elucidation of these mechanisms would be very useful. Baicalin needs to be transformed into baicalein by enzymes in the intestine before exerting its biological activity, since baicalein has a higher bioavailability [13]. In addition, baicalein has better lipid solubility than wogonin, which allows it to cross a cell membrane and more easily exert its biological activity within cells [14]. When studying TCM monomers, Bui et al. confirmed that baicalein can reduce airway inflammation and airway remodeling in asthmatic mice by reversing the T helper 1 cell (Th1)/Th2 cytokine imbalance and releasing histamine from mast cells [15]. Another study showed that the classic inflammation pathway, nuclear factor-kappa B (NF-κB) pathway, is inhibited by baicalein, thereby reducing airway inflammation and airway remodeling in asthma [16]. The above evidence demonstrates that baicalein has a clear biological purpose, and has great potential to be of value in the treatment of asthma. However, baicalein is a multi-target drug, and there is still a lack of systematic research on its use for the treatment of asthma.

In recent years, with the rapid development of systems biology, bioinformatics, and network analysis, network pharmacology has emerged as a promising approach in drug discovery and development. It integrates various disciplines, such as pharmacology, bioinformatics, and network analysis, to explore the complex interactions between drugs and targets at the molecular level. One of the key applications of network pharmacology is in the identification of potential drug targets and active compounds for the treatment of various diseases, including asthma [17]. However, the in-silico prediction of potential targets and compounds must be followed by experimental validation to confirm their efficacy and safety. Molecular docking is a computational tool used to study the interactions between a small molecule and a target protein [18]. It can predict the binding affinity and orientation of a ligand to the active site of the protein, providing valuable insights into the mechanistic action of a drug. Thus, the integration of network pharmacology, molecular docking, and experimental validation provides a powerful tool for drug development [19].

**Fig. 1:**
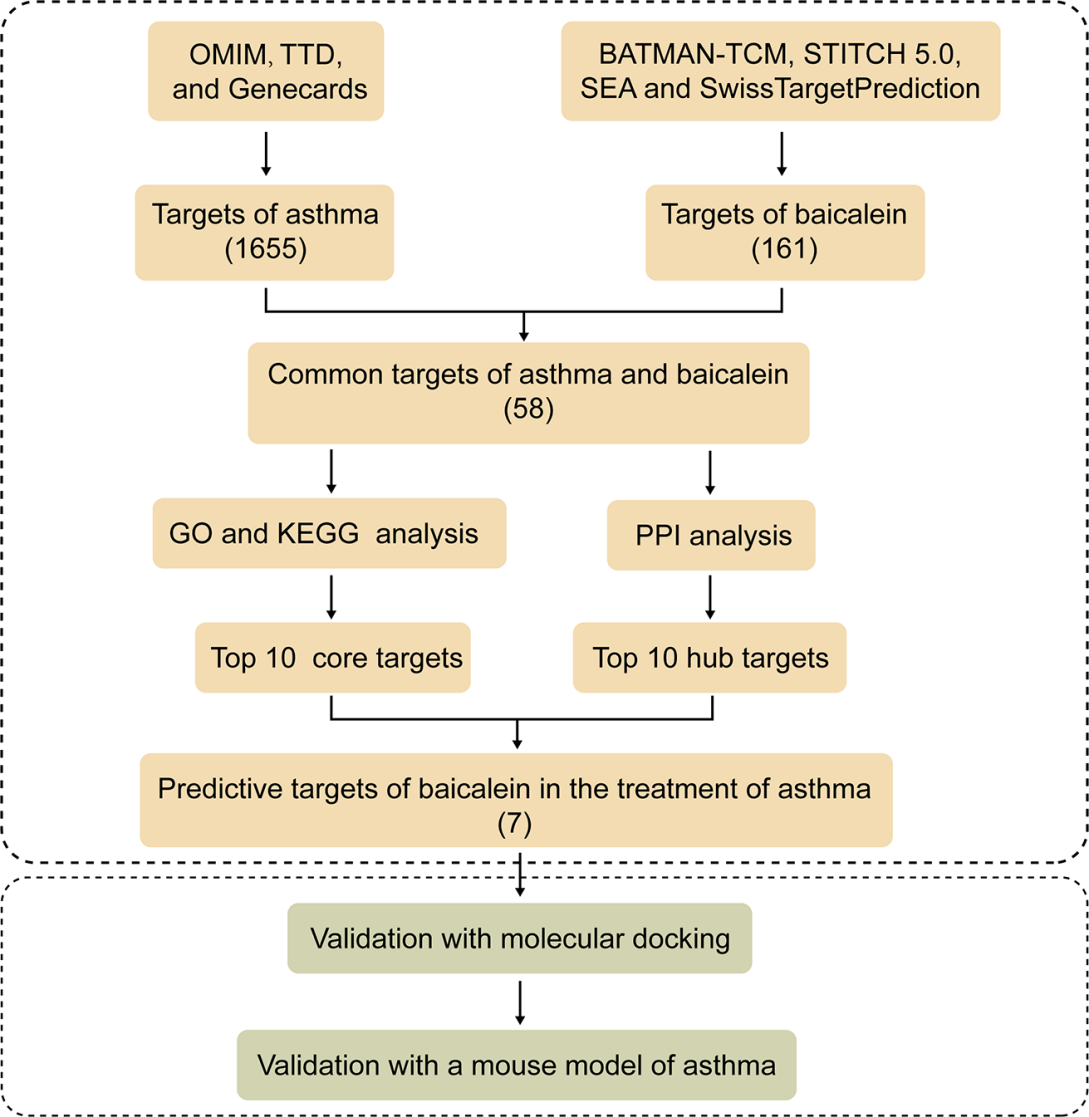
Flowchart of the analysis and validation

In our study, we utilized network pharmacology to predict potential targets for treating asthma with baicalein, followed by molecular docking to preliminarily verify the binding ability between the predicted targets and baicalein. Finally, we ran live experiments to verify the therapeutic efficacy and targets of baicalein in an OVA-induced asthma mouse model. Our results provide new and more comprehensive evidence for the use of baicalein in the development of asthma drugs. The research flowchart is shown in Fig. 1.

## Materials and methods

### Targets of asthma

To identify potential targets for asthma, we conducted a search for the keyword “asthma” in the gene maps of various databases, including the Online Mendelian Inheritance in Man (OMIM, https://omim.org/), the therapeutic target database (TTD, http://db.idrblab.net/ttd/), and Genecards (https://www.genecards.org/). Any targets that were duplicated across multiple databases were eliminated to ensure that our list only contained unique candidates.

### Targets of baicalein

We employed several bioinformatics tools to identify molecular targets of baicalein, using the keyword “baicalein” as the search term. These tools included the Bioinformatics Analysis Tool for Molecular Mechanism of Traditional Chinese Medicine (BATMAN-TCM, http://bionet.ncpsb.org/batman-tcm/), Search Tool for Interactions of Chemicals 5.0 (STITCH5.0, http://stitch.embl.de/), Similarity Ensemble Approach (SEA, https://sea.bkslab.org/), and SwissTargetPrediction (http://www.swisstargetprediction.ch/).

To obtain a compound-target-pathway network, we used the PubChem CID (5281605) of baicalein in BATMAN-TCM. In SwissTargetPrediction, SEA, and STITCH, we used either the Simplified Molecular Input Line Entry System (SMILES) or the term “baicalein” to identify related targets in Homo sapiens.

### Gene ontology (GO) and Kyoto encyclopedia of genes and genomes (KEGG) analyses

To gain a better understanding of the function and signaling pathways associated with the intersecting genes, we converted the gene symbols to gene IDs (EntrezID) and analyzed the results visually using the “clusterProfiler” package in R. Furthermore, we performed Gene ontology (GO) analysis to explore the molecular function (MF), biological process (BP), and cellular component (CC) of these genes in relation to asthma. Additionally, we conducted KEGG analysis to investigate the major anti-asthma signaling pathways linked with the intersecting genes. We selected the top 20 KEGG pathways and generated a target-pathway network using Cytoscape 3.9.0 as described in a previous study [20].

### Protein–Protein Interaction (PPI) Network map

We utilized the STRING database (https://string-db.org/) to create a protein-protein interaction (PPI) network, which enabled us to identify potential target gene interactions based on co-expression, fusion, neighborhood, and co-localization. Our analysis was conducted specifically for Homo sapiens, and the resulting network comprised nodes that represented proteins, while the edges represented functional associations between potential targets.

The PPI network was imported into Cytoscape 3.9.0 for further analysis and visualization, and we employed CytoHubba to identify hub genes. These hub genes are proteins with a high degree of connectivity in the network, and they are thought to be critical in regulating the network’s behavior.

### Molecular docking

To assess the binding affinity and stability of baicalein with predicted targets, we conducted molecular docking using Autodock Vina. We retrieved the crystal structures of seven protein targets, including RAC-alpha serine/threonine-protein kinase (AKT1, PDB ID: 1UNQ), endothelial growth factor A (VEGFA, PDB ID: 1MKK), epidermal growth factor receptor (EGFR, PDB ID: 6TG0), proto-oncogene tyrosine-protein kinase (SRC, PDB ID: 2SRC), mitogen-activated protein kinase 3 (MAPK3, PDB ID: 4QTB), matrix metalloproteinase-9 (MMP9, PDB ID: 4XCT), and mitogen-activated protein kinase 1 (MAPK1, PDB ID: 6SLG), from the RCSB Protein Data Bank (https://www.rcsb.org/). We optimized the structures by removing water molecules and adding hydrogen atoms. The default values of the docking run options and the Genetic Algorithm were used. We obtained the binding scores and visualized the 2D and 3D results with the highest scores, using Ligplot 2.2.5 and PyMoL, respectively.

### Experimental animals and treatments

Six-week-old male BALB/c mice were obtained from Slca Laboratory Animal Co. Ltd. (Hunan, China) and were housed under controlled conditions with a 12-hour light/dark cycle, free access to food and water, and constant room temperature. All animal protocols were approved by the Xiangya Hospital Institutional Ethics Committee at Central South University, China (No. 202,201,001).

The mice were randomly divided into four groups: a control (CTRL) group, an ovalbumin (OVA) group, a low dose baicalein (10 mg/kg/d, intraperitoneal injection) group(OVA BAI 10), and a high dose baicalein (50 mg/kg/d, intraperitoneal injection) group(OVA BAI 50). On days 0, 7, and 14, mice were sensitized by intraperitoneal injection with 100 µg OVA (Sigma-Aldrich #A5503) and 2 mg Al(OH)_3_ (Sigma-Aldrich #239,186) dissolved in 200 µl saline. From days 21–25, mice were exposed to 5% OVA (Sigma-Aldrich #A5253) aerosol for 20 min per day. In the baicalein treatment groups, the corresponding dose of baicalein (Sigma-Aldrich #465,119) was administered by intraperitoneal injection 30 min before the atomized-OVA exposure. In the control group, all drugs were replaced with an equal volume of saline. In the OVA group, baicalein treatment was replaced by an equal volume of solvent (5% DMSO, 45% PEG200, and 50% saline). Mice were euthanized 24 h after the final OVA exposure, and their lungs were harvested for further analysis.

### Histological staining

After anesthesia with 2% pentobarbital sodium (60 mg/kg), the mice were subjected to three lung perfusions with cold phosphate buffer saline (PBS). The left lungs were then removed, fixed in 4% paraformaldehyde for 24 h at 4 °C, and embedded in paraffin. Thin sections (5 μm) were obtained and stained with hematoxylin and eosin (H&E) or Masson’s trichrome staining. The inflammation score was based on a previously established method, and was calculated according to the degree of vascular/parabronchial infiltration, bronchial lumen exudation, and lung parenchyma infiltration (score range, 0–26) [21, 22]. The degree of airway remodeling was assessed by measuring the amount of collagen using Image-J software (National Institutes of Health 1.8.0_112).

### Immunohistochemistry

Paraffin sections of lung tissue were dewaxed and hydrated before undergoing antigen retrieval. Endogenous tissue peroxidases were eliminated by incubating the sections in 3% H_2_O_2_ for 25 min. The sections were then blocked with 10% goat serum for 30 min, followed by incubation with primary antibodies (α-SMA, #GB111364, 1:2000, Servicebio) at 4 °C overnight. The sections were then incubated with secondary antibodies (#GB23303, 1:200, Servicebio) for 1 h at room temperature and counterstained with hematoxylin. These sections were examined using an A1 light microscope (Zeiss, Jena, Germany), and three randomly selected fields of view (×20 magnification) were captured for each section. The thickness of the smooth muscle cell ring was calculated using Image-J software (National Institutes of Health 1.8.0_112).

### Enzyme-linked immunosorbent assay (ELISA)

As per the provided instructions, levels of IL-4, IL-13, and IFN-γ in lung tissue, as well as the level of serum IgE, were determined using the supplied assay kits (FY Biotech #FY2165, #FY2173, #FY2182, #FY2056).

### Flow cytometry assay

Cells were collected from bronchoalveolar lavage fluid (BALF) and pre-incubated with Fc-blocking anti-mouse CD16/32 antibody (BD Pharmingen, #553,141) before the staining process. Subsequently, dead cells were excluded by DAPI staining (BD Pharmingen, #564,907). The cells then underwent surface staining with anti-mouse CD45 (BD Pharmingen, #561,037), anti-mouse CD11b (BD Pharmingen, #561,688), anti-mouse GR-1 (BD Pharmingen, #561,103), anti-mouse CD-11c (BD Pharmingen, #561,022), and anti-mouse MHCII (Biolegend, #107,613) for 30 min at 4 °C. All samples were analyzed using Cytek Dxp Athena flow cytometer, and the data were processed using FlowJo software (version 10). The method for eosinophil count was conducted following established protocols from previous study [23], and is illustrated in Fig. S1. Initially, live leukocytes were selected based on the expression of CD45 and exclusion of DAPI, effectively removing debris, erythrocytes, and dead cells. Subsequently, lymphocytes were differentiated through the analysis of the SSC-A/CD11b plot, while neutrophils were identified using the SSC-A/GR1 plot among the diverse cell populations. Ultimately, eosinophils were isolated from the remaining cells using the MHC-II/CD11c plot.

### Real-time quantitative polymerase chain reaction (RT-qPCR)

Total RNA was extracted from lung tissues using TRIzol (Vazyme #R401-01), followed by reverse transcription of 1 µg RNA using Takara reverse transcription reagents (Takara #RR047A). Real-time qPCR was performed using an Applied Biosystems instrument (Thermo Fisher Scientific #Quantstudio 7 Flex) using HieffqPCR SYBR Green Master Mix (Yeasen #11202ES08), and the expression of mouse *Akt1, Vegfa, Egfr, Src, Mapk3, Mmp9, Mapk1*, and *Actb* was measured. The experimental protocols strictly followed the manufacturer’s instructions. Gene expression levels were analyzed using the 2-ΔΔCt method and normalized to the housekeeping gene *Actb*. Gene-specific primers listed in Table S1were used for mRNA quantification.

### Western blotting

Total protein was extracted from lung tissue, and 20 µg of protein was separated by 10% polyacrylamide gel electrophoresis and transferred onto Polyvinylidene fluoride (PVDF) membranes (Millipore). The membranes were blocked with 5% non-fat milk in TBS-T buffer (50mM Tris-HCl, pH 7.5, 150mM NaCl, 0.1% Tween-20) for 1 h at room temperature, followed by overnight incubation with primary antibodies against extracellular signal-regulated kinase (ERK, 1:1000, #4695, Abmart), phosphorylated ERK (p-ERK,1:1000, #4370, Abmart), VEGFA (1:1000, #A12303, Abclonal), EGFR (1:1000, #4267S, Cell Signaling Technology), and Glyceraldehyde 3-phosphate dehydrogenase (GAPDH,1:1000, #5519, Cell Signaling Technology) at 4 °C. After washing with TBS-T buffer, the membranes were incubated with secondary antibodies (Goat Anti-Rabbit IgG H&L(HRP) 1:20000, #511,203, Zen-bioscience) for 1 h at room temperature. The protein bands were detected using an enhanced chemiluminescence detection system, and visualized with a Bio-Rad Chemi Doc XRS imaging system. The molecular weights of the target proteins were confirmed using pre-stained protein markers. The protein expression levels were quantified by densitometry using ImageJ software and normalized to the GAPDH loading control.

### Statistical analysis

The statistical analysis of the data was performed using GraphPad Prism 8 software (GraphPad Software, La Jolla, CA, USA). The results were presented as mean ± standard error of the mean (SEM). Normal and equally distributed data were analyzed using one-way ANOVA followed by Dunn’s post hoc analyses, while non-normally distributed data were analyzed using Kruskal-Wallis test. A p-value less than 0.05 was considered statistically significant.

## Results

### Fifty-eight common targets of asthma and baicalein and their functional annotations

**Fig. 2:**
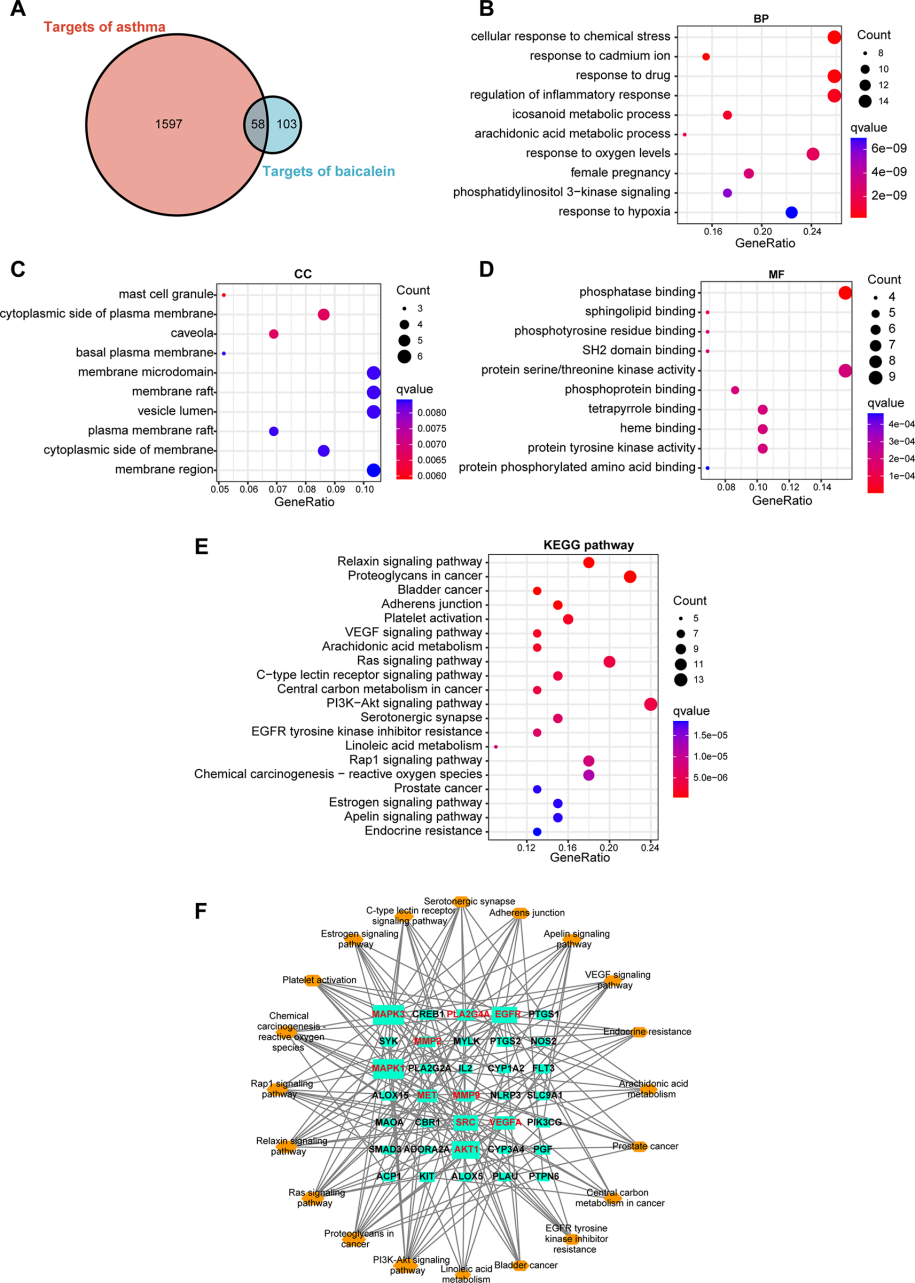
Screening and functional annotation of disease drug targets. (**A**) The Venn diagram of potential targets of asthma and baicalein. (**B**) Top 10 biological processes (BP) identified by GO analysis. (**C**) Top 10 cellular components (CC) identified by GO analysis. (**D**) Top 10 molecular functions (MF) identified by GO analysis. (**E**) Top 20 pathways from KEGG analysis. (**E**) Top 20 KEGG pathways network. The genes highlighted in red indicate the top 10 highest scoring targets

We obtained 134, 185, and 1503 asthma-related targets from the TTD, OMIM, and Genecards databases, respectively. Similarly, we collected 11, 10, 104, and 77 baicalein-related targets from the BATMAN, STITCH, SwissTargetPrediction, and SEA databases, respectively. After removing duplicates and merging search results, a total of 1655 asthma-related targets and 161 baicalein-related targets were obtained. Furthermore, by taking the intersection of the compound target genes and disease-related genes, we identified 58 common targets of baicalein and asthma (Fig. 2A, Table S2).

GO analysis was used to understand the biological functions of the 58 common targets. A total of 1302 GO entries were found to be significantly enriched (p < 0.05), including 1202 molecular processes, 28 cellular components, and 72 molecular functions (Table S3). The 10 most significant entries in terms of BP, CC, and MF are shown in Fig. 2B–D. The results indicate that baicalein may regulate a range of biological processes, including cellular response to chemical stress, response to cadmium ion, response to drugs, regulation of inflammatory response, icosanoid metabolic process, arachidonic acid metabolic process, response to oxygen levels, female pregnancy, and phosphatidylinositol 3-kinase signaling. The main cellular components involved were mast cell granule, caveola, cytoplasmic side of plasma membrane, cytoplasmic side of membrane, plasma membrane raft, vesicle lumen, membrane raft, membrane microdomain, basal plasma membrane, and membrane region. Finally, the main molecular functions identified were phosphatase binding, sphingolipid binding, protein tyrosine kinase activity, heme binding, tetrapyrrole binding, phosphoprotein binding, protein serine/threonine kinase activity, SH2 domain binding, phospho-tyrosine residue binding, and protein phosphorylated amino acid binding.

We further conducted a KEGG enrichment analysis to identify pathways that were significantly enriched. A total of 123 KEGG pathways were found to be significantly enriched (p < 0.05, Table S4), with the 20 most enriched pathways shown in Fig. 2E. These pathways included proteoglycans in cancer, relaxin signaling pathway, bladder cancer, adherens junction, platelet activation, VEGF signaling pathway, arachidonic acid metabolism, ras signaling pathway, C-type lectin receptor signaling pathway, central carbon metabolism in cancer, PI3K-Akt signaling pathway, serotonergic synapse, EGFR tyrosine kinase inhibitor resistance, rap1 signaling pathway, linoleic acid metabolism, chemical carcinogenesis - reactive oxygen species, apelin signaling pathway, estrogen signaling pathway, prostate cancer, and endocrine resistance. To understand the relationship between the top 20 significantly enriched pathways and the genes involved in these pathways, we constructed a gene pathway network. This network consisted of 43 nodes (20 pathways and 35 genes) and was analyzed topologically. The resulting network diagram identified the top-10 degree genes, including MAPK3, MAPK1, AKT1, EGFR, SRC, VEGFA, mesenchymal-epithelial transition factor (MET), Phospholipase A2 Group IVA (PLA2G4A), MMP9, and MMP2 (Fig. 2F, Table S5). These genes are the core targets of baicalein treatment against asthma.

### PPI network analysis reveals predictive targets for baicalein in the treatment of asthma

**Fig. 3:**
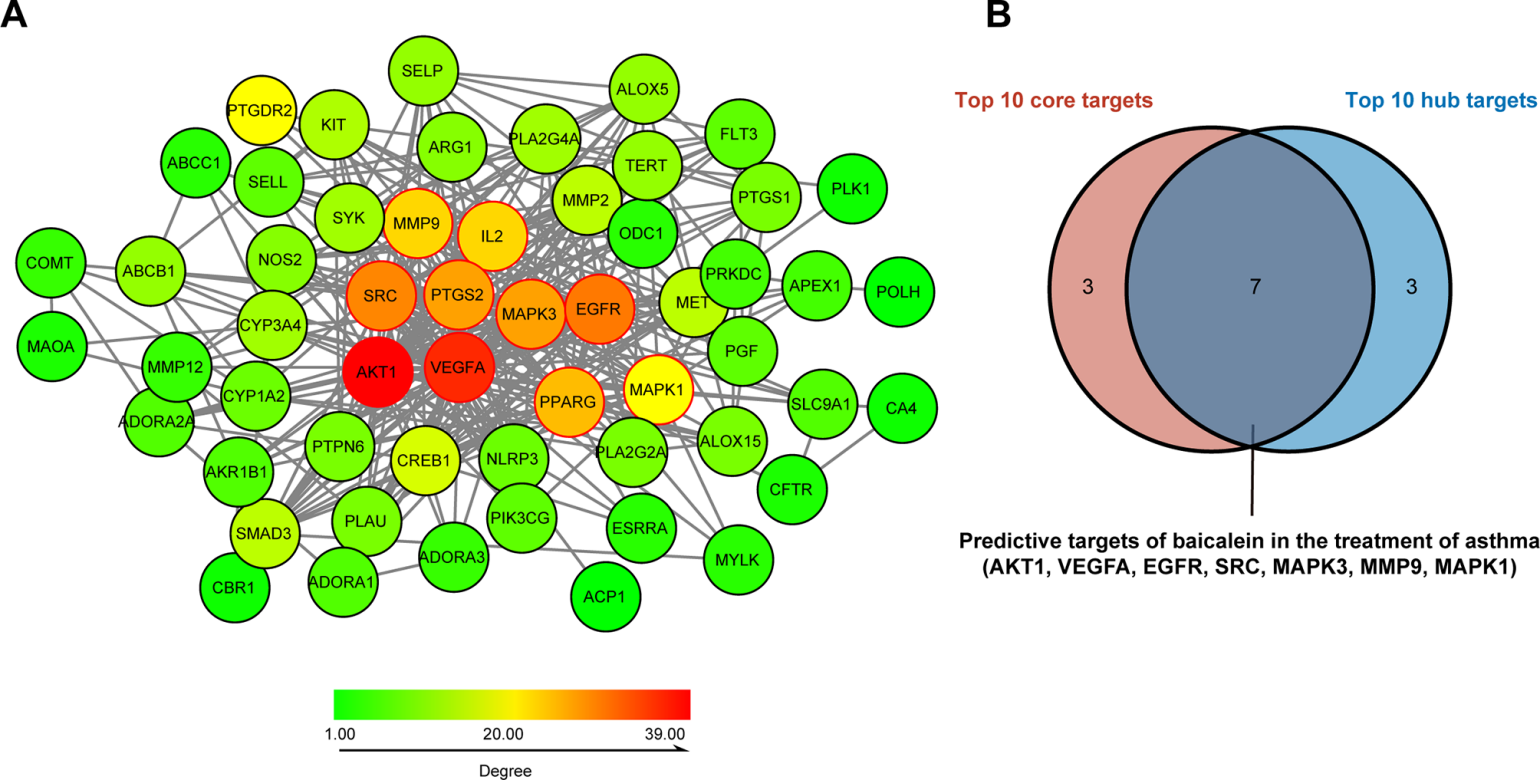
Protein-protein interaction network and secondary prediction targets screening. (**A**) Protein-protein interaction network. The genes enclosed by the red border represent the top 10 genes with the highest score. (**B**) The Venn diagram illustrates the overlap between core and hub targets. Core targets represent the top 10 genes with the highest degree values in the KEGG pathway interaction network, while hub genes refer to the top 10 genes with the highest MMC values in the PPI network

To identify the hub genes of baicalein anti-asthma, we constructed a PPI network. An overall view of the relationships within the 58 targets is presented in Fig. 3A. Based on the results of MCC topology analysis, we selected the 10 genes with the highest MCC values including: AKT1, VEGFA, EGFR, SRC, Prostaglandin-endoperoxide synthase 2 (PTGS2), MAPK3, Peroxisome proliferator-activated receptor gamma (PPARG), MMP9, IL2, MAPK1, which were considered to be the top 10 hub genes (Table S6). The stability of the predicted targets of baicalein was ensured in terms of metabolic pathways and protein interactions. We took the intersection of the 10 genes with the highest values in the KEGG pathway interaction network and the 10 genes with the highest MMC values in the PPI network. Finally, we obtained 7 predicted targets of baicalein for asthma treatment, namely AKT1, VEGFA, EGFR, SRC, MAPK3, MMP9, MAPK1 (Fig. 3B).

### Baicalein exhibits strong binding affinity to 7 predicted targets

**Fig. 4:**
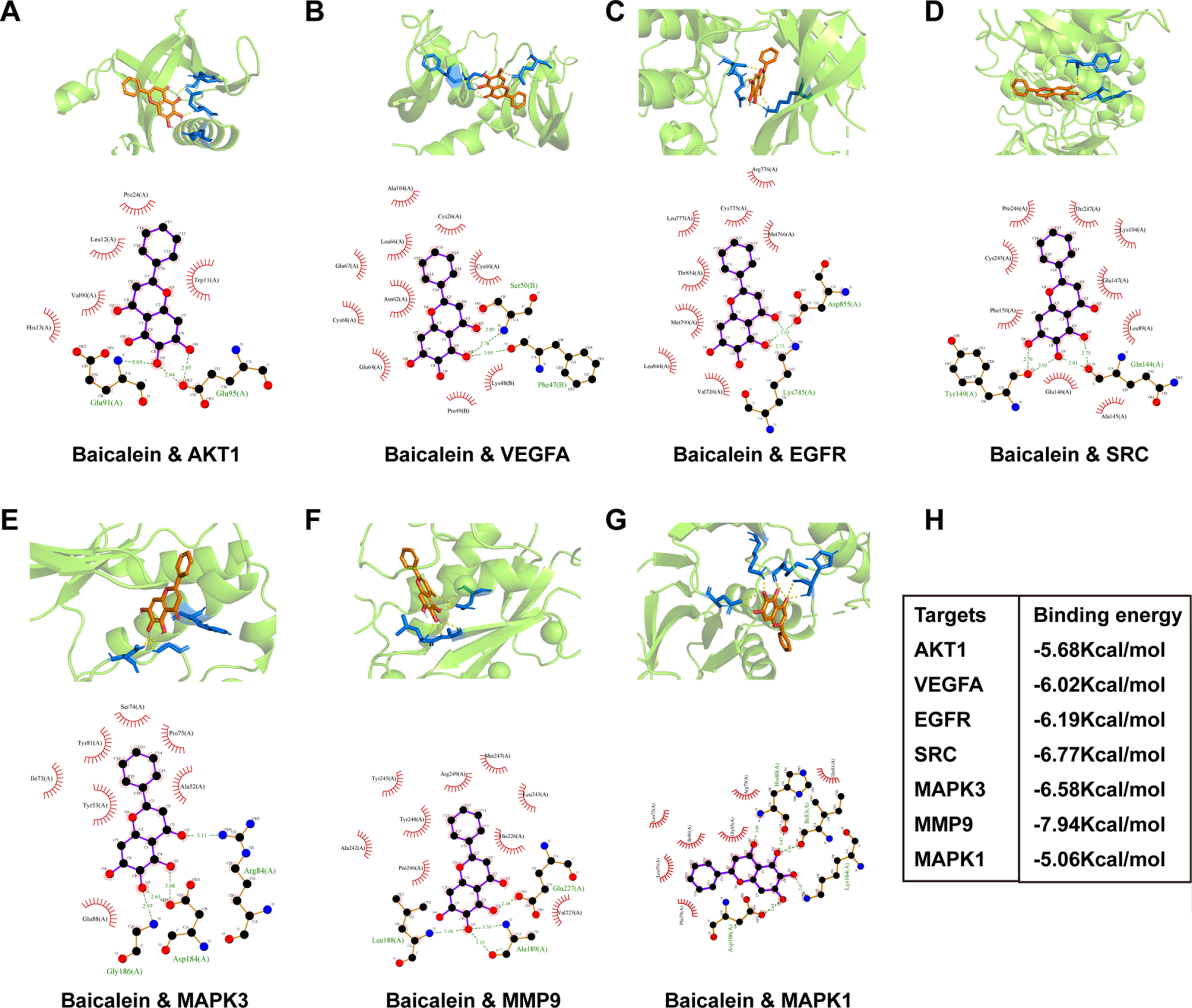
Molecular docking betweenbaicalein and the seven predicted targets. (**A**–**G**)The 2D and 3Dinteraction diagrams of baicalein and the seven predicted targets. (**H**) Molecular docking binding energy

We conducted molecular docking of baicalein with the 7 predicted targets and generated 2D and 3D representations of the docking results (Fig. 4A–G). These showed that baicalein had a binding energy less than − 5 kcal/mol with all 7 predicted targets (Fig. 4H, Table S7), indicating that baicalein has a strong binding affinity and high biological activity towards these targets.

### Baicalein alleviated airway inflammation and airway remodeling in OVA-induced asthmatic mice

**Fig. 5:**
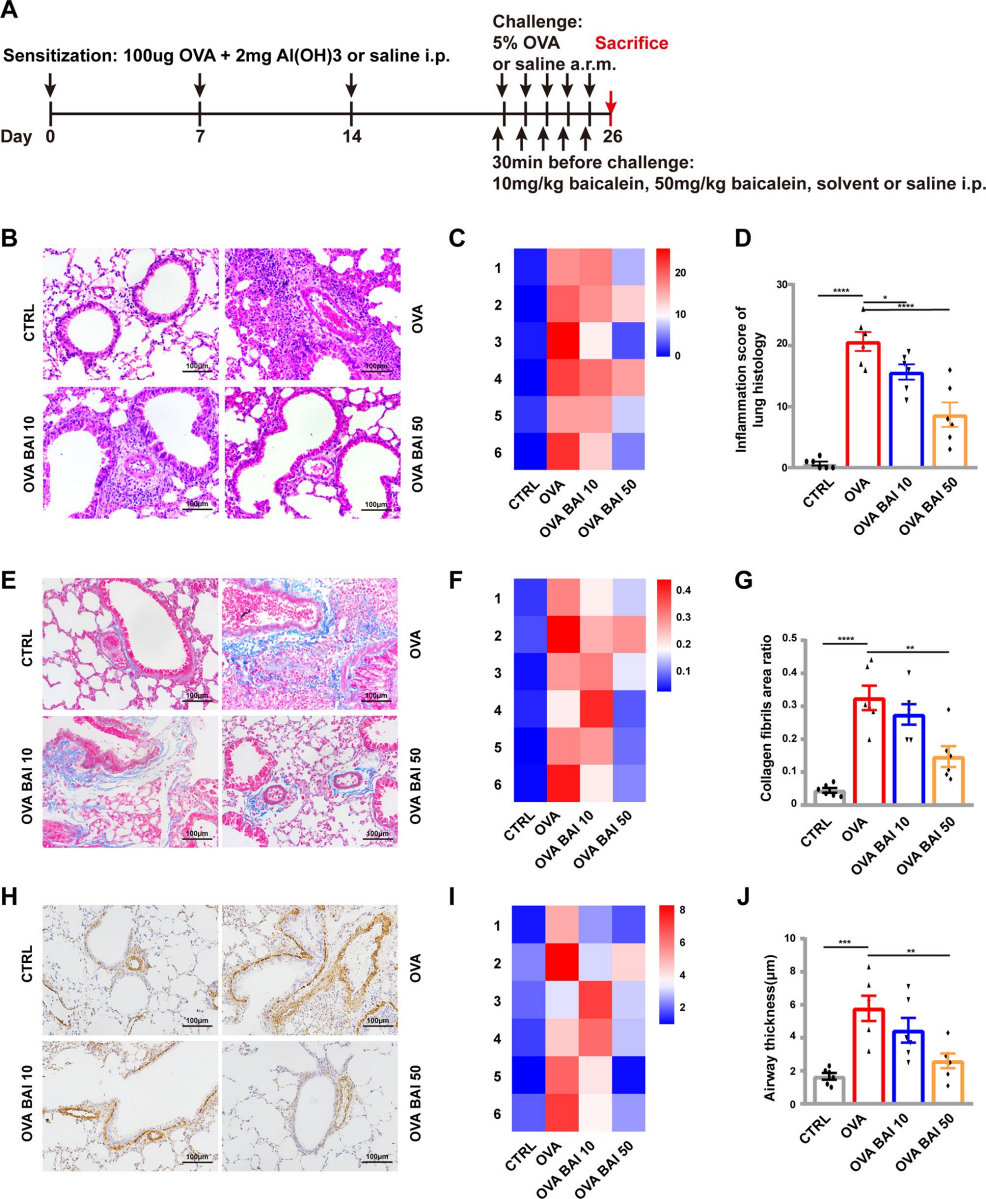
Efficacy of baicalein in reducing airway inflammation and airway remodeling in asthmatic mice. (**A**) Modeling of asthma and a baicalein intervention plan. (**B**) H&E staining of mouse lung tissue. (**C**) Heat map of inflammatory infiltration score. (**D**) Statistical analysis of inflammatory infiltration score. (**E**) Masson staining of mouse lung tissue. (**F**) Heat map of collagen deposition percent (**G**) Statistical analysis of collagen deposition percent. (**H**) Immunohistochemistry of α-SMA. (**I**) Heat map of smooth muscle thickness (**G**) Statistical analysis of smooth muscle thickness. Scale bar length indicates 100 μm, n = 6, mean ± SEM, *p < 0.05, **p < 0.01, ***p < 0.001 and ****p < 0.0001 vs. the OVA group

To evaluate the therapeutic effects of baicalein on airway inflammation and airway remodeling in asthma, we administered intraperitoneal injections of baicalein in an OVA-induced asthma model as described above (Fig. 5A). We observed significant infiltration of inflammatory cells around the bronchi in the OVA model group, the OVA BAI 10 group showed only a slight reduction in infiltration, and the OVA BAI 50 group showed further improvement in reducing inflammatory cell infiltration compared with the OVA group (Fig. 5B). We scored each mouse for inflammation and found that the majority (4/6) of mice in the OVA BAI 50 group had scores less than 10, while all mice in the OVA group had scores over 10 (Fig. 5C). Statistical analysis confirmed that the OVA BAI 50 group had significantly lower inflammation scores compared to the OVA group (p < 0.0001, Fig. 5D). Additionally, we detected and quantified collagen deposition around the airway, as a hallmark of airway remodeling. Compared to the CTRL group, a large amount of collagen deposition was found in the OVA group, while the OVA BAI 50 group showed a significant reduction relative to the OVA group (Fig. 5E). We found that collagen deposition was greater than 20% in 83.33% (5/6) of mice in the OVA group, while only 16.67% (1/6) of mice in the OVA BAI 50 group had this level of deposition (Fig. 5F). The OVA BAI 50 group showed significantly lower collagen deposition than the OVA group (p < 0.01, Fig. 5G). Airway smooth muscle hyperplasia is another marker of airway remodeling, and immunohistochemical results showed that airway smooth muscle thickness was thicker in the OVA model group than in the CTRL group, but decreased in the OVA BAI 50 group compared with the OVA model group (Fig. 5H). We measured smooth muscle thickness using imageJ and found that it was approximately 2 μm in the CTRL group, more than 4 μm in 83.33% (5/6) of the OVA group, and less than 4 μm in 83.33% (5/6) of the OVA BAI 50 group (Fig. 5I). Statistical analysis revealed that smooth muscle thickness was significantly increased in the OVA group compared to the CTRL group(p < 0.001), and significantly lower in the OVA BAI 50 group compared to the OVA group (p < 0.01, Fig. 5J).

### Baicalein inhibited the OVA-induced Th2 immune response

**Fig. 6:**
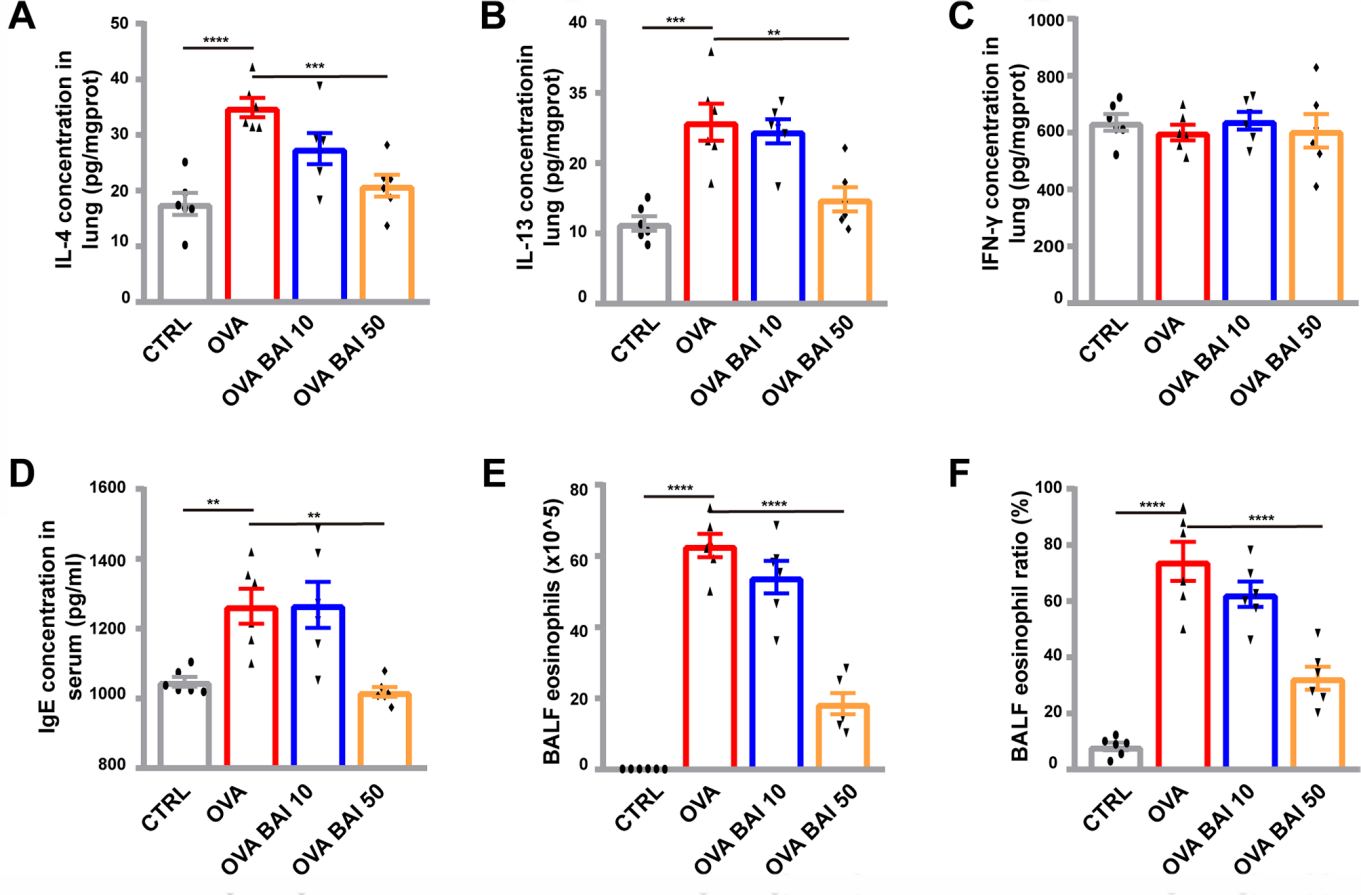
Characterization of Th2 inflammation following baicalein treatment. (**A–C**) IL-4, IL-13 and INF-γ levels in lung tissue homogenate. (**D**) IgE level in serum. (**F**) BALF eosinophil count. (**F**) BALF eosinophil ratio. n = 6, mean ± SEM, **p < 0.01, ***p < 0.001 and ****p < 0.0001 vs. the OVA group

The ELISA results showed that levels of Th2 inflammatory factors, such as lung IL-4 and IL-13, were significantly elevated in the OVA group compared to the CTRL group (p < 0.001, p < 0.0001), whereas in the OVA BAI 50 group, these levels were significantly reduced compared to the OVA group (p < 0.001, p < 0.01), and were similar to those observed in the CTRL group (Fig. 6A, B). Additionally, the levels of peripheral blood IgE, BALF eosinophil count and ratio were significantly higher in the OVA group compared to the CTRL group (p < 0.01, p < 0.0001,p < 0.0001), but were significantly reduced in the OVA BAI 50 group compared to the OVA group (p < 0.01, p < 0.0001,p < 0.0001,Fig. 6D F). Interestingly, no significant alterations in the Th1 inflammatory factor IFN-γ were observed in any of the four groups (Fig. 6C).

### Validation of predicted target gene expression changes in OVA asthmatic mice following baicalein treatment

**Fig. 7:**
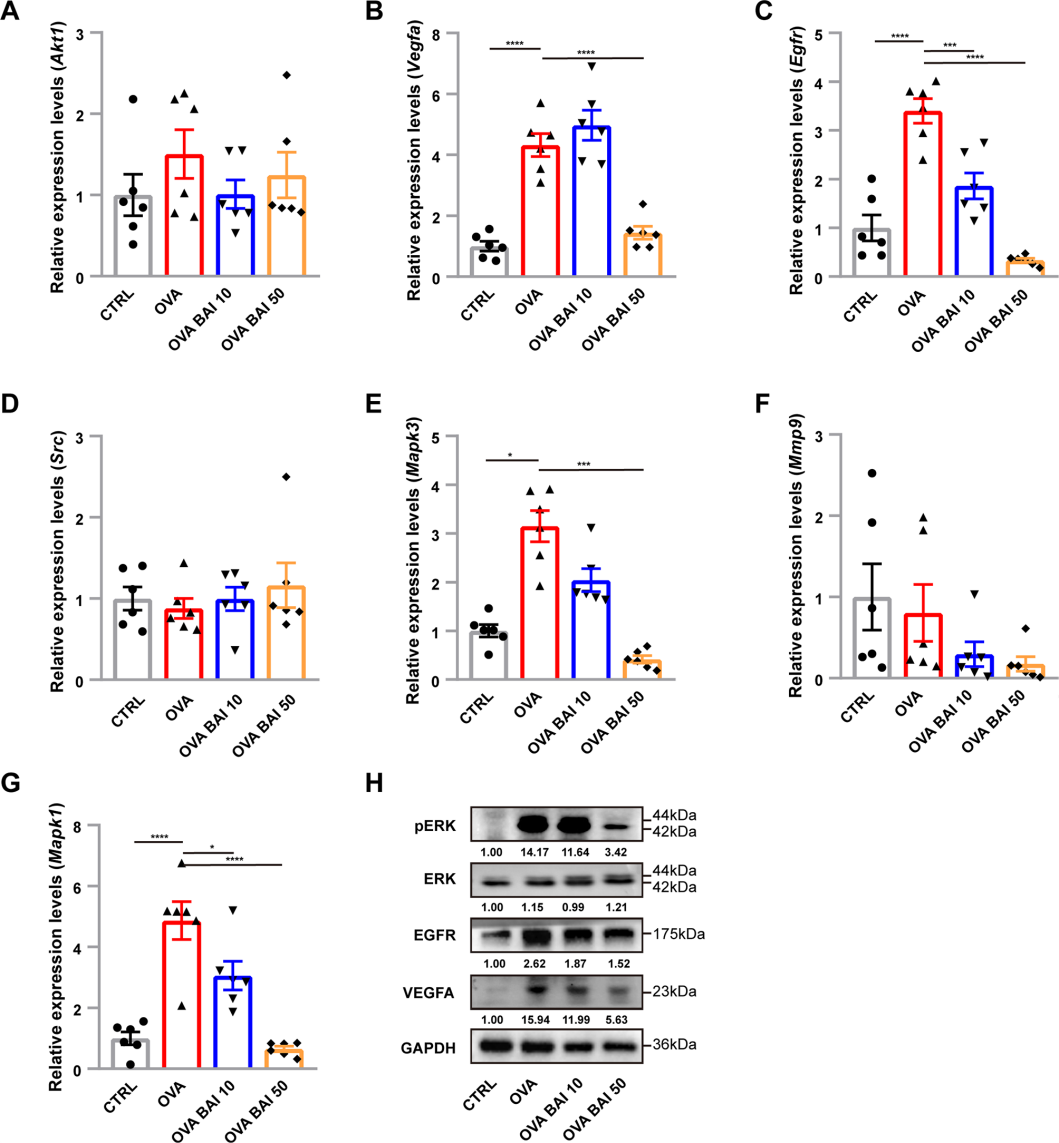
Effect of baicalein on VEGF and EGFR signaling pathways. (**A**–**G**) The relative expression level of seven predicted genes detected by RT-qPCR. n = 6, mean ± SEM, *p < 0.05,***p < 0.001 and****p < 0.0001 vs. the OVA group. (**H**) Protein expression representation of target molecule. The values below the bars represent the mean of the ratios relative to the expression of the target proteins in the CTRL group and were pre-normalized with GAPDH, n = 3

The expression levels of *Vegfa, Egfr, Mapk3*, and *Mapk1* genes were found to be significantly up-regulated in the OVA group compared to the CTRL group, and their expression was significantly reversed by baicalein in the OVA BAI 50 group, as determined by RT-qPCR analysis. No significant differences in the expression of *Akt1, Src*, and *Mmp9* genes were observed among the four groups (Fig. 7A G). To further validate the changes in expression at the protein level, we performed Western Blotting for EGFR and VEGFA, two genes with differential transcript levels, as well as for the phosphorylated form of ERK protein (pERK), a downstream effector of the MAPK signaling pathway. The results showed that the expressions of EGFR and VEGFA were significantly upregulated in the OVA model, and the pERK level was significantly increased. In contrast, in the OVA BAI 50 group, these trends were significantly reversed (Fig. 7H). These findings suggest that baicalein may exert its therapeutic effect on asthma by inhibiting the VEGF and EGFR signaling pathways.

## Discussion

In our study, we employed a network pharmacology approach to prioritize seven predicted targets (AKT1, VEGFA, EGFR, SRC, MAPK3, MMP9, MAPK1) of baicalein for asthma treatment, and confirmed its high binding affinity to these targets through molecular docking. More importantly, we showed the efficacy of baicalein in reducing airway inflammation and airway remodeling in an OVA-induced asthma mouse model and verified the involvement of the VEGF and EGFR signaling pathways in the therapeutic effect of baicalein.

TCM has been widely used for the treatment of asthma based onto its long history and potential therapeutic benefits. Several studies have shown that TCM treatments such as Scutellaria baicalensis [24]and icariin [25]can improve lung function and reduce the frequency and severity of asthma attacks. However, the mechanism underlying how TCM ingredients for treating asthma work, is not well understood, which highlights the need for more effective methods to accelerate the understanding of the mechanisms that explain how treatments such as TCM succeed. Network pharmacology has emerged as a powerful tool for identifying potential therapeutic targets and understanding the underlying mechanisms of TCM in treating specific diseases. Compared to traditional laboratory screening methods, network pharmacology is faster, more cost-effective, and more reliable [26]. In our study, we used network pharmacology to identify 7 predicted targets that may be involved in the treatment of asthma out of the 161 baicalein targets obtained from the BATMAN, STITCH, SwissTargetPrediction, and SEA databases. Molecular docking was then performed to predict the binding affinity and stability of baicalein with these predicted targets [27]. We found that all seven predicted targets formed stable complexes with baicalein, which aided in the design of subsequent experimental validation studies.

We used an OVA-induced asthma mouse model to verify that the expression of VEGFA, EGFR and the phosphorylation of ERK are involved in the pathogenesis of asthma, and that baicalein can inhibit the expression of VEGFA, EGFR and the phosphorylation of ERK in these mice. Furthermore, in the KEGG analysis, we found that the VEGF and EGFR signaling pathways ranked 6th and 13th, respectively, in terms of significant enrichment. These findings are consistent with previous studies, Lee et al. found that increased airway smooth muscle hyperplasia and collagen deposition were found in asthmatic mice overexpressing VEGF, and that VEGF overexpression enhanced IL-4 and IL-13 production and eosinophilic airway inflammation in asthmatic mice [28]. Zhang et al. showed that porcine epithelial-derived factors can attenuate OVA-induced airway inflammation and airway remodeling in mice by inhibiting VEGF expression in lung tissue [29]. Deng et al. found that chrysin alleviates house dust mite induced eosinophilic asthma by targeting the Src/EGFR signaling pathway [30].

VEGF is involved in multiple physiological and pathological processes, such as angiogenesis, vascular permeability, and inflammatory response [31]. In the pathogenesis of asthma, increased vascular permeability in the airway is an important factor leading to airway edema and mucus secretion. Studies have shown that VEGF polymorphism is positively correlated with airway hyperresponsiveness and the severity of asthma [32], and that VEGF can exacerbate airway inflammation by promoting the migration and activation of inflammatory cells [33]. Therefore, inhibition of the expression and function of VEGF may be a promising strategy for the treatment of asthma.

EGFR is a transmembrane receptor belonging to the receptor tyrosine kinase family, which plays a significant role in the regulation of cell proliferation, differentiation, and survival [34]. It is expressed in various cell types, including airway epithelial cells [35], smooth muscle cells [36], and immune cells [37]. Dysregulation of EGFR signaling has been implicated in the pathogenesis of asthma. Several studies have shown that EGFR is overexpressed in the airway epithelium of asthmatic patients and animal models of asthma, leading to increased airway inflammation, airway hyperresponsiveness, and airway remodeling [30, 38, 39]. EGFR activation can also trigger the release of pro-inflammatory cytokines, such as IL-6 and IL-8, and the production of mucus in the airways [40]. The role of EGFR inhibitors in asthma treatment has been investigated in several studies. Preclinical studies have demonstrated that EGFR inhibitors, including erlotinib and gefitinib, can decrease airway inflammation and airway hyperresponsiveness in animal models of asthma [30, 41, 42]. However, clinical studies of EGFR inhibitors for asthma have not been reported.

The limitations of this study include: Firstly, reversible airflow limitation is an important pathological feature of asthma [43], but due to experimental conditions, we were unable to evaluate this parameter when assessing the therapeutic efficacy of baicalein. Secondly, the key molecules involved in the phosphorylation of VEGFA, EGFR, and ERK, as well as their upstream and downstream molecules, are still unclear. Future studies will focus on enhancing the efficacy evaluation of baicalein treatment for asthma, and also, we need to use molecular-specific inhibitors to fully elucidate the signaling mechanisms involved. Thirdly, the OVA mouse model represents just one state of asthma. The efficacy of baicalein needs to be validated in additional models, such as the dust mite-induced model, cockroach extract-induced model, and lipopolysaccharide-induced model. Finally, the safety of baicalein as a therapeutic agent requires further testing.

## Conclusions

Our study utilized network pharmacology and molecular docking to predict the potential targets of baicalein in the treatment of asthma. The in vivo experiments conducted in this study confirmed the predicted targets and showed that baicalein has therapeutic effects in reducing airway inflammation and airway remodeling in asthma. Our results also demonstrated that the therapeutic effects of baicalein are mediated through the inhibition of the VEGF and EGFR signaling pathways, and suppression of Th2 cytokine production. These findings provide new insights that will help in the development of baicalein-based therapies for the treatment of asthma, and highlights the potential of network pharmacology and molecular docking in drug discovery and development.

### Electronic supplementary material

Below is the link to the electronic supplementary material.

**Supplementary Material 1:** **Table S1.** Primer Sequences Used for RT-qPCR

**Supplementary Material 2:** **Table S2.** Degree of Gene pathway network

**Supplementary Material 3:** **Table S3.** Gene Ontology

**Supplementary Material 4:** **Table S4.** KEGG pathway

**Supplementary Material 5:** **Table S5.** The affinity of the baicalein with core targets

**Supplementary Material 6:** **Table S6.** Scores of PPI network by cytoHubba

**Supplementary Material 7:** **Table S7.** Target gene information

**Supplementary Material 8:** **Figure S1.** Eosinophil Screening Process. Initially, viable leukocytes were chosen by their expression of CD45 and the exclusion of DAPI, ensuring the removal of debris, erythrocytes, and deceased cells. Following this, the distinction of lymphocytes was accomplished through analysis of the SSC-A/CD11b plot, while neutrophils were discerned using the SSC-A/GR1 plot within the heterogeneous cell populations. Finally, eosinophils were separated from the remaining cells utilizing the MHC-II/CD11c plot

## Data Availability

The data supporting the current study’s findings are available from the corresponding author upon reasonable request.
