# Supplementary figures and images for: VEGF and EGFR signaling pathways are involved in the baicalein attenuation of OVA-induced airway inflammation and airway remodeling in mice
Source: Respir Res. 2024 Jan 4;25:10. doi: 10.1186/s12931-023-02637-6 (PMC10765748; doi:10.1186/s12931-023-02637-6)

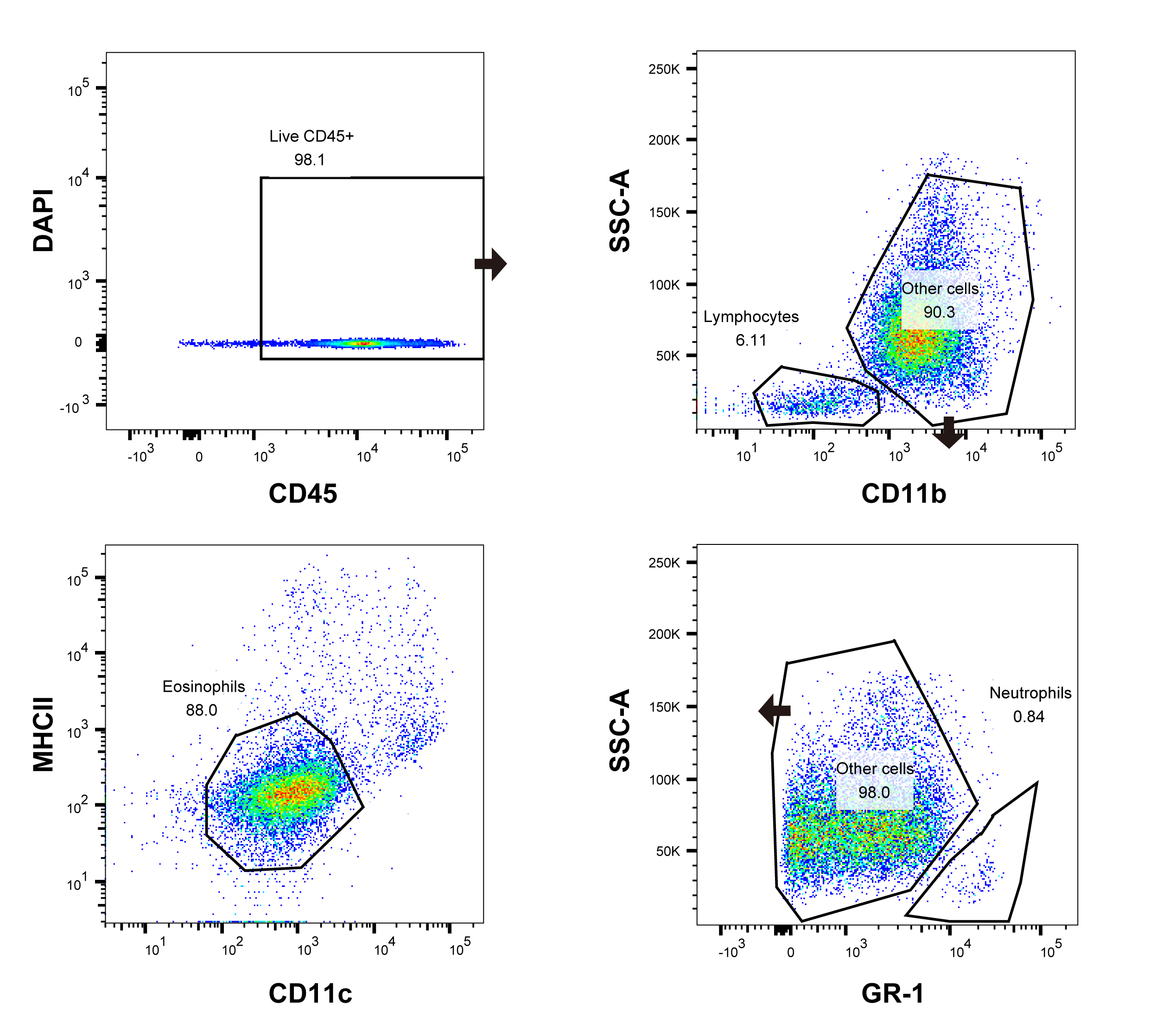

Supplement: Supplementary file 8 — Supplementary Material 8: Figure S1. Eosinophil Screening Process. Initially, viable leukocytes were chosen by their expression of CD45 and the exclusion of DAPI, ensuring the removal of debris, erythrocytes, and deceased cells. Following this, the distinction of lymphocytes was accomplished through analysis of the SSC-A/CD11b plot, while neutrophils were discerned using the SSC-A/GR1 plot within the heterogeneous cell populations. Finally, eosinophils were separated from the remaining cells utilizing the MHC-II/CD11c plot [file 12931_2023_2637_MOESM8_ESM.tif]
